# Postpartum Depression and Subsequent Autoimmune Diseases in Taiwan

**DOI:** 10.3390/ijerph15081783

**Published:** 2018-08-20

**Authors:** Chien-Yu Lin, Cheng-Kai Li, Jui-Ming Liu, Ren-Jun Hsu, Heng-Chang Chuang, Fung-Wei Chang

**Affiliations:** 1 Department of Pediatrics and Infectious Disease, Hsinchu MacKay Memorial Hospital, Hsinchu City 300, Taiwan; mmhped.lin@gmail.com; 2 Department of Gynecology, Taoyuan General Hospital, Ministry of Health and Welfare, Taoyuan 330, Taiwan; 4985@mail.tygh.gov.tw; 3 Division of Urology, Department of Surgery, Taoyuan General Hospital, Ministry of Health and Welfare, Taoyuan 330, Taiwan; mento1218@gmail.com (J.-M.L.); chuang20110617@yahoo.com.tw (H.-C.C.); 4 Graduate Institute of Life Sciences, National Defense Medical Center, Taipei 114, Taiwan; hsurnai@gmail.com; 5 Biobank Management Center of the Tri-Service General Hospital, National Defense Medical Center, Taipei 114, Taiwan; 6 Department of Pathology and Graduate Institute of Pathology and Parasitology, the Tri-Service General Hospital, National Defense Medical Center, Taipei 114, Taiwan; 7 Department of Obstetrics & Gynecology, Tri-Service General Hospital, National Defense Medical Center, Taipei 114, Taiwan; 8 Superintendent, Tri-Service General Hospital Penghu Branch, National Defence Medical Center, Penghu Branch, Magong City 880, Taiwan

**Keywords:** postpartum depression, autoimmune disease, pregnancy, pernicious anemia, rheumatoid arthritis, Graves’ disease, national health insurance database

## Abstract

Postpartum depression (PPD) is one of the most common examples of postnatal morbidity, but the subsequent risks of autoimmune diseases in patients with PPD have yet to be fully investigated. This nationwide population-based study utilized data of the National Health Insurance Research Database of Taiwan for the period from 1996 to 2013. In total, 45,451 women with primiparity were identified. Among them, 542 patients with PPD were enrolled as a study group while 2165 matched patients without PPD were enrolled as a control group. The demographic characteristics and comorbidities of the patients were analyzed, and Cox regression analysis was applied to calculate the hazard ratios for the risk of autoimmune diseases. Of the 2707 women enrolled in this study, 469 (17.3%) patients with newly diagnosed autoimmune diseases were identified, including 123 (22.7%) in the PPD group and 346 (16%) in the non-PPD group. After adjusting for confounding factors, it was determined that the patients with PPD had a significantly higher risk of subsequent autoimmune diseases (adjusted hazard ratio (aHR): 1.61, 95% confidence interval (CI): 1.30–1.99; *p* < 0.001). Specifically, increased risks of pernicious anemia (aHR: 3.85, 95% CI: 2.06–7.22), rheumatoid arthritis (aHR: 2.62, 95% CI: 1.28–5.39), and Graves’ disease (aHR: 1.57, 95% CI: 1.05–2.33) were observed in the PPD group. This study demonstrated that patients with PPD have higher risks of subsequent autoimmune diseases, especially pernicious anemia, rheumatoid arthritis, and Graves’ disease. This useful information provides physicians with clues regarding the associations between autoimmune diseases and PPD.

## 1. Introduction

Postpartum depression (PPD) is a major morbidity during the perinatal period. Approximately 9% of women who have given birth suffer from PPD, a condition which has consequences for both a mother and her neonates [1]. The prompt diagnosis and treatment of PPD, however, can protect the health and quality of life of all family members. Multiple risk factors for PPD have been identified, including demographic, clinical, psychosocial, husband/marriage-related, and child-related factors [2,3]. The frequency of primiparity is higher in patients with PPD. However, the exact pathophysiology of PPD is not fully understood. Dramatic changes in hormones and extreme mood swings are believed to contribute to PPD. In addition, immune-mediated cytokine alterations have been found to play important roles in the underlying mechanism of PPD [4,5,6]. A possible link between PPD and the cytokine-hormone axis has also been reported [7]. However, the entire pathogenesis of PPD remains unclear.

Autoimmune diseases are disorders arising from abnormal immune responses to a normal body part, and more than eighty kinds of autoimmune diseases have been identified. The estimated incidence of autoimmune diseases ranges from 3.2–9.4%, and the incidences and prevalence have increased significantly over the last 30 years [8,9]. Environmental factors affect the risk of autoimmune diseases, and the pathogeneses of the various individual diseases differ [10,11,12]. Autoimmune diseases are significantly more prevalent in females, and sex hormones may contribute to this significant gender predilection [13]. Overwhelming hormone changes occur during pregnancy, and increased risks of autoimmune diseases have been observed in pregnant women [14]. A significantly higher risk of autoimmune diseases in the first year after delivery has also been reported [15]. As such, it is believed that women have a different level of overall risk for such diseases during the perinatal period. 

Both PPD and autoimmune diseases are associated with hormonal changes, and both are prevalent during the perinatal period. An increased risk of autoimmune thyroid disease has also been noted in women with PPD (with a 19% risk found for such women versus a 5% in a control group) [16]. As such, it is possible that a link exists between PPD and autoimmune diseases. Therefore, we conducted this retrospective nationwide population-based study to investigate the association between PPD and autoimmune diseases.

## 2. Materials and Methods 

### 2.1. Data Source and Collection 

This study was approved by the Institutional Review Board of the Tri-Service General Hospital (approval number: TSGHIRB NO B-104-21.). We conducted this study using data from National Health Insurance Research Database (NHIRD) of Taiwan. The National Health Insurance program is the unique medical insurance system of Taiwan that covers 99.5% of Taiwan’s 23 million residents [17]. Diagnoses included in the NHIRD are categorized according to the International Classification of Diseases, 9th revision, Clinical Modification (ICD-9-CM) coding system, with all details regarding patient diagnoses and treatment-related information being included in the database [18]. The Longitudinal Health Insurance Database 2000 (LHID2000) is a sub-dataset of the NHIRD and is comprised of the data for one million people randomly selected from the NHIRD in 2000 [19]. We explored the LHID2000 and extracted information for further analysis. 

### 2.2. Study Population 

**Figure 1:**
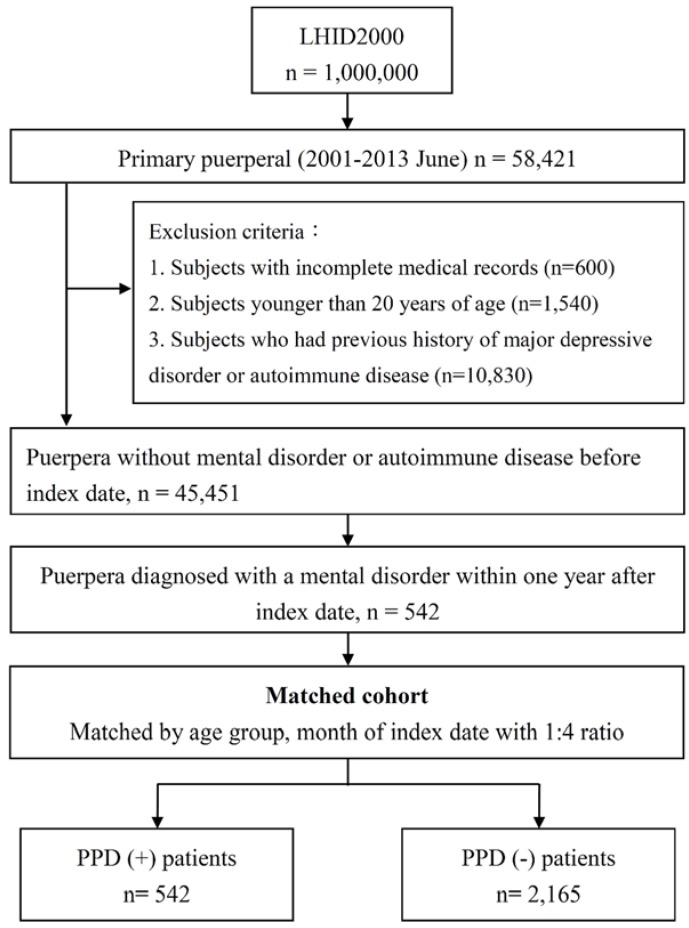
The flow chart for enrollment of study cohorts.

We selected participants by using the LHID2000 data covering the period from January 1996 through December 2013. The flow chart for enrollment is shown in Figure 1. First, we identified the women with primiparity included in the LHID2000 (n = 58,421). Next, the following exclusion criteria were applied: (1) Subjects with incomplete medical records (n = 600), (2) Subjects younger than 20 years of age (n = 1540), (3) Subjects who had previous history of major depressive disorder or autoimmune disease (n = 10,830). Under Taiwanese civil law, only people who have reached twenty years of age are considered adults. We chose to include only adult women in this study with the approval of the Institutional Review Board. The index date for each participant referred to the date of delivery, and any diagnosis of depression within one year after delivery was defined as PPD (ICD-9-CM code: 296.2, 296.3, 296.5, 296.82, 300.4, 309.0, 309.1, 311, 648.4, 648.44, 780.79) Any diagnosis of major depressive disorder was made by psychiatrists who prescribed antidepressants for treatment. Women with PPD were enrolled as the PPD group (n = 542). Moreover, for each patient in the PPD group, approximately four participants matched for age and comorbidities were included in a non-PPD control group (i.e., the control group had an n = 2165). We then tracked both groups to identify those who were newly diagnosed with autoimmune diseases after the index date. The detailed diagnoses of autoimmune diseases and corresponding ICD-9-CM codes were summarized in Table S1.

### 2.3. Study Outcomes and Covariates

The key outcome was the number of newly diagnosed autoimmune diseases, with those diagnoses being made by rheumatologists or dermatologists. The incidence rate (IR) for each of the individual autoimmune diseases was also calculated. Censoring was defined as death, the dates of diagnoses of autoimmune diseases, or the end of the follow-up period. We further analyzed several variables for both groups, including age, monthly income, geographic area of residence, urbanization level of residence, and comorbidities. Age was divided into 5 groups based on five-year intervals: 20 to 24 years, 25 to 29 years, 30 to 34 years, 35 to 39 years, and ≥40 years. The monthly incomes of the study participants were recorded in New Taiwan Dollars (NTD) and categorized into the following 4 income levels: <NTD 20,000, NTD 20,000 to NTD 39,999, NTD 40,000 to NTD 59,999, and ≥NTD 60,000. The different geographic areas of residence in Taiwan were divided into 4 areas: the northern region, central region, southern region, and other region (eastern and outlying islands). The urbanization levels of residence in Taiwan were classified into 4 categories. Comorbidities included diabetes mellitus, hypertension, hyperlipidemia, coronary artery disease, stroke, alcoholism, obesity (ICD-9-CM: 278), and tobacco use disorder.

### 2.4. Statistical Analysis 

Student’s *t*-test and the Chi-square test were used to analyze and compare the categorical demographic characteristics, including age, income, geographic area of residence, level of urbanization of residence, and comorbidities, of the PPD group and the control group. We also determined the incidence rates of the individual autoimmune diseases for both groups. Cox proportional hazards regressions were used to evaluate the relationships between PPD and subsequent autoimmune diseases, and hazard ratios (HRs) with 95% confidence intervals (CIs) were calculated. Further adjustment for potential confounders (including age, gender, income, geographic area of residence, level of urbanization of residence, and comorbidities) was performed in all models, and adjusted HRs (aHRs) were calculated. A two-sided *p* value <0.05 was viewed as the threshold for statistical significance. The statistical analyses were performed using the SPSS software version 19.0 (SPSS Inc., Chicago, IL, USA) and Microsoft^®^ SQL Server^®^ 2008 software (Microsoft Unternehmen, Redmond, DC, USA) for data management.

## 3. Results

In total, 58,421 women with primiparity were identified (Figure 1). There were 2707 study subjects who met all the inclusion and exclusion criteria; 542 of those subjects had PPD, while the other 2165 had no PPD and served as the control group. The demographic data of both groups are summarized in Table 1. In terms of age, women aged 25–29 years and 30–34 years suffered most commonly from PPD. In terms of income, those in the lowest income category suffered most commonly from PPD. Moreover, women with PPD most commonly lived in the Northern part of Taiwan and in areas with the highest level of urbanization. Hyperlipidemia, tobacco use, hypertension, and diabetes mellitus were leading comorbidities in patients with PPD. There were no significant differences in age, income, geographic distribution, or urbanization levels between the PPD group and the control group. However, the rates of various comorbidities were significantly higher in the PPD group compared with the non-PPD group.

Overall, 469 (17.3%) of the women had at least one kind of autoimmune disease. The associations between PPD and the various autoimmune diseases (in which total n ≥ 5 in the PPD group) as determined by Cox regression analysis are shown in Table 2 in the PPD group, 123 (22.7%) of the women had autoimmune disease with 3222.7 person-years. The IR was 38.2 per 1000 person-years. In the non-PPD group, autoimmune diseases occurred in 346 (16%) of the women with 13686.5 person-years. The IR was 25.3 per 1000 person-years. The women with PPD had a higher overall risk of subsequent autoimmune diseases with an aHR of 1.61 (95% CI: 1.30–1.99, *p* < 0.01). Compared with the non-PPD group, the incidence rates for each of the individual autoimmune diseases were higher in the PPD group, especially those for RA, Graves’ disease, and pernicious anemia. After adjusting for confounding factors, it was found that the women with PPD had significantly higher risks of RA (aHR: 2.62, 95% CI: 1.28–5.39, *p* < 0.05), Graves’ disease (aHR: 1.57, 95% CI: 1.05–2.33, *p* < 0.05), and pernicious anemia (aHR: 3.85, 95% CI: 2.06–7.22, *p* < 0.01) than the non-PPD group.

## 4. Discussion

PPD is an important health threat during the perinatal period; however, research studies investigating the subsequent risk of autoimmune diseases are scarce. In this large-scale cohort study, we found an increased overall risk of autoimmune diseases in women with PPD (aHR 1.61), including significantly higher risks of subsequent pernicious anemia (aHR: 3.85), RA (aHR: 2.62), and Graves’ disease (aHR: 1.57). Physicians should thus remain aware of the potential for autoimmune diseases in patients with a history of PPD. 

Autoimmune diseases are complex disorders with multiple pathogenic factors, and the pathogeneses of these diseases are still not completely understood. Females have a higher risk of autoimmune diseases than males, and sex hormones play important roles in the pathogenic mechanisms of autoimmune diseases [20,21,22]. During the peripartum period, women are more susceptible than otherwise to several kinds of autoimmune diseases and psychiatric disorders [2,15,23]. Women experience dramatic physiological changes during the perinatal period. Considerable increases and decreases in the levels of various sex hormones and changes in immunologic profiles are well documented [5,20,24]. Serum IL-6 and leukemia inhibitory factor receptor levels are significantly higher in pregnant women at the end of pregnancy than in nonpregnant women [5]. For women who have an autoimmune disease and subsequently become pregnant, pregnancy can induce amelioration of the mother’s disease, such as occurs in RA, while exacerbating or having no effect on other autoimmune diseases like systemic lupus erythematosus [14]. Estrogen and testosterone are potential physiological regulatory factors for the peripheral development of CD4 + CD25 + T regulatory cells. As such, the modulation of estrogen in females (both pre-menopausal and post-menopausal) and testosterone in males can be used to treat stress-related immune imbalances that result in autoimmune diseases in both sexes [20]. Alterations of these hormones contribute to inflammatory responses in the central nervous system and increase the risk of postpartum psychiatric disorders [23]. For example, CSF IL-6 and tumor necrosis factor (TNF)-alpha levels have been found to be significantly higher during the peripartum period [4]. From this viewpoint, immune mechanisms may play a role in the etiopathology of postpartum depressive mood shifts, and PPD may be regarded as a kind of autoimmune diseases [25]. Individuals with one of the autoimmune diseases have a higher risk of developing other kinds of autoimmune diseases. Patients with PPD may thus have different levels of susceptibility to other autoimmune diseases, and the results of this study demonstrated that women with PPD do, in fact, have a higher overall risk of subsequent autoimmune diseases.

Pernicious anemia is caused by immune-mediated chronic atrophic gastritis and the malabsorption of vitamin B12 followed by destruction of parietal cells and autoantibodies against intrinsic factors [26]. Pernicious anemia is associated with many systemic diseases and is regarded as part of polyglandular autoimmune syndrome type 2, which includes such disorders as autoimmune thyroid disease, Addison’s disease, type 1 DM, and vitiligo [27]. In animals with autoimmune gastritis, IL-21 and TNF-alpha are highly expressed [28]. Increased levels of TNF-alpha have also been noted in women with PPD. Furthermore, decreased vitamin B12 levels, which have also been noted in pernicious anemia, have been reported during the peripartum period [29]. Moreover, women in Iran with anemia (hemoglobin < 11 g/dl) were found to have a 4.64 times increased risk of PPD [30]. A link between PPD and pernicious anemia may thus exist, and this study also demonstrated a higher risk of pernicious anemia in women with PPD. Therefore, the aggressive evaluation of anemia and timely supplementation of vitamin B12 in women with PPD are suggested. 

This study also found a significantly increased risk of RA in patients with PPD. The association between RA and PPD may be bidirectional. In patients with RA, depression is more common and is associated with poorer RA outcomes [31,32]. In the Chinese adult population, the prevalence of depression is 48% in patients with RA, and depression is an important comorbid disease [33]. Similarly, sex hormones are thought to play crucial roles in the pathophysiology of RA, and approximately 75% of pregnancies are accompanied by amelioration of RA signs and symptoms in women with RA [14,21]. Further studies are required, however, to explore the detailed mechanism connecting PPD and RA. Meanwhile, physicians should pay attention to the symptoms and signs of RA in women with PPD.

Drastic changes in thyroid hormones during pregnancy are well established, and up to 23% of all new mothers experience postpartum thyroid dysfunction [34]. An association between PPD and Graves’ disease has also been noticed in several studies [16,35,36,37]. For example, in a Danish nationwide population-based cohort study, women with first-onset postpartum psychiatric disorders had a higher risk of autoimmune thyroid diseases than women without psychiatric disorders (incidence rate ratio = 2.16, 95% CI: 1.45–3.20) [35]. Although the underlying etiologies connecting PPD and Graves’ diseases have not been completely elucidated, evaluations of thyroid function in women with first-onset PPD are suggested [38]. The value of individualized management of PPD and comorbid thyroid disease in postpartum women is also reinforced by the results of this study [39]. 

This nationwide population-based cohort study showed an increased risk of autoimmune diseases in women with PPD, and the benefits of analyzing the large population-based database analyzed herein were demonstrated. However, the study also had some limitations. First, detailed laboratory data are not included in the NHIRD, such that the levels of sex hormones, inflammatory markers, T regulatory cells, pivotal deregulated cells, and other immune profiles could not be investigated. Second, the severities of PPD and autoimmune diseases were not evaluated, and the relative risks may be different in patients with different severities. Furthermore, antidepressants have been found to have immunomodulatory effects that could potentially affect the incidences of autoimmune diseases [40]. Lastly, this was a retrospective study, so further prospective studies are warranted to clarify the relationship between PPD and autoimmune diseases. 

## 5. Conclusions

In conclusion, this large-scale nationwide population-based study found that women with PPD have higher risks of subsequent autoimmune diseases, especially pernicious anemia, RA, and Graves’ disease. Physicians should thus strive to be aware of the symptoms and signs of autoimmune diseases in patients with PPD. In patients with PPD and anemia, the prompt evaluation of pernicious anemia and the timely supplementation of vitamin B12 are valuable. A survey of RA should be initiated in any patients with PPD presenting with the symptoms and signs of RA. Screenings of thyroid function are also suggested for women with PPD.

## Figures and Tables

**Table 1:** Clinical and demographic characteristics of the study subjects.

		Number of Individuals		
		PPD	Without PPD	
Variable		n = 542	n = 2165	*p* Value
**Age Group**				1
	20–24	100 (18.5%)	400 (18.5%)	
	25–29	191 (35.2%)	762 (35.2%)	
	30–34	171 (31.5%)	684 (31.6%)	
	35–39	71 (13.1%)	284 (13.1%)	
	≥40	9 (1.7%)	35 (1.6%)	
**Income Group**				0.43
	<20,000	398 (73.4%)	1476 (68.2%)	
	20,000–39,999	116 (21.4%)	535 (24.7%)	
	40,000–59,999	27 (5.0%)	132 (6.1%)	
	≥60,000	1 (0.2%)	22 (1.0%)	
**Geography**				0.40
	North	293 (54.1%)	1142 (52.7%)	
	Central	93 (17.2%)	392 (18.1%)	
	South	137 (25.2%)	580 (26.8%)	
	Other (eastern and outlying islands)	19 (3.5%)	51 (2.4%)	
**Urbanization**				0.66
	1 (highest)	262 (48.4%)	987 (45.6%)	
	2	133 (24.5%)	560 (25.9%)	
	3	101 (18.6%)	438 (20.2%)	
	4 (lowest)	46 (8.5%)	180 (8.3%)	
**Comorbidity**				
	DM	30 (5.5%)	62 (2.9%)	<0.05
	Hypertension	35 (6.5%)	79 (3.6%)	<0.05
	Hyperlipidemia	49 (9.0%)	142 (6.6%)	<0.05
	CAD	23 (4.2%)	35 (1.6%)	<0.001
	Stroke	13 (2.4%)	13 (0.6%)	<0.001
	Alcoholism	21 (3.9%)	12 (0.6%)	<0.001
	Obesity	9 (1.7%)	28 (1.3%)	0.51
	Tobacco use disorder	36 (6.6%)	82 (3.8%)	<0.05

DM, Diabetes mellitus. CAD, Coronary artery disease.

**Table 2:** The associations between PPD and autoimmune diseases (in which total n ≥ 5 in the PPD group) as determined by Cox regression analysis.

	PPD Group (n = 542)			Without PPD Group (n = 2165)			Crude HR	Adjusted HR *
Autoimmune Disease	n (%)	PY	IR	n (%)	PY	IR	(95% CI)	(95% CI)
All	123 (22.7)	3222.7	38.2	346 (16.0)	13686.5	25.3	1.50 (1.22 to 1.84) ‡	1.61 (1.30 to 1.99) ‡
RA	13 (2.4)	3809.0	3.4	21 (1.0)	15358.7	1.4	2.49 (1.25 to 4.97) †	2.62 (1.28 to 5.39) †
Psoriasis	13 (2.4)	3804.4	3.4	44 (2.0)	15256.7	2.9	1.18 (0.64 to 2.20)	1.19 (0.63 to 2.23)
Graves’ disease	35 (6.5)	3689.8	9.5	93 (4.3)	15007.8	6.2	1.53 (1.04 to 2.26) †	1.57 (1.05 to 2.33) †
Crohn disease	23 (4.2)	3730.8	6.2	81 (3.7)	15016.7	5.4	1.14 (0.72 to 1.82)	1.31 (0.82 to 2.09)
Pernicious anemia	19 (3.5)	3785.0	5.0	22 (1.0)	15347.9	1.4	3.49 (1.89 to 6.45) ‡	3.85 (2.06 to 7.22) ‡
Hereditary hemolytic anemia	12 (2.2)	3794.9	3.2	32 (1.5)	15291.1	2.1	1.51 (0.78 to 2.93)	1.75 (0.90 to 3.41)
Alopecia areata	6 (1.1)	3857.6	1.6	12 (0.6)	15410.2	0.8	2.00 (0.75 to 5.32)	1.97 (0.72 to 5.37)

† *p* < 0.05 for comparison between patients with two groups; ‡ *p* < 0.001 for comparison between patients with two groups; * Each variable was adjusted for gender, age, income, geography, urbanization, and comorbidity; PPD, postpartum depression; PY, person-years; IR, incidence rate per 1000 person-years; RA, rheumatoid arthritis.

## Supplementary Materials

The following are available online at http://www.mdpi.com/1660-4601/15/8/1783/s1, Table S1: Diseases and corresponding ICD-9-CM codes in the study.

## References

[1] Vesga-Lopez O., Blanco C., Keyes K., Olfson M., Grant B.F., Hasin D.S. (2008). Psychiatric disorders in pregnant and postpartum women in the United States. Arch. Gen. Psychiatry.

[2] Mehta S., Mehta N. (2014). An Overview of Risk Factors Associated to Post-partum Depression in Asia. Ment Illn..

[3] Silverman M.E., Reichenberg A., Savitz D.A., Cnattingius S., Lichtenstein P., Hultman C.M., Larsson H., Sandin S. (2017). The risk factors for postpartum depression: A population-based study. Depression Anxiety.

[4] Boufidou F., Lambrinoudaki I., Argeitis J., Zervas I.M., Pliatsika P., Leonardou A.A., Petropoulos G., Hasiakos D., Papadias K., Nikolaou C. (2009). CSF and plasma cytokines at delivery and postpartum mood disturbances. J. Affect. Disord..

[5] Maes M., Lin A.H., Ombelet W., Stevens K., Kenis G., De Jongh R., John C., Eugène B. (2000). Immune activation in the early puerperium is related to postpartum anxiety and depressive symptoms. Psychoneuroendocrinology.

[6] Nemeroff C.B. (2008). Understanding the pathophysiology of postpartum depression: Implications for the development of novel treatments. Neuron.

[7] Stoner R., Camilleri V., Calleja-Agius J., Schembri-Wismayer P. (2017). The cytokine-hormone axis—The link between premenstrual syndrome and postpartum depression. Gynecol. Endocrinol..

[8] Cooper G.S., Bynum M.L.K., Somers E.C. (2009). Recent Insights in the Epidemiology of Autoimmune Diseases: Improved Prevalence Estimates and Understanding of Clustering of Diseases. J. Autoimmun..

[9] Lerner A., Jeremias P., Matthias T. (2015). The World Incidence and Prevalence of Autoimmune Diseases is Increasing. Int. J. Celiac Dis..

[10] Bach J.F. (2018). The hygiene hypothesis in autoimmunity: The role of pathogens and commensals. Nat. Rev. Immunol..

[11] Cooper G.S., Stroehla B.C. (2003). The epidemiology of autoimmune diseases. Autoimmun. Rev..

[12] Liu J.M., Chiu F.H., Lin C.Y., Chang F.W., Hsu R.J. (2017). Incidence of autoimmune diseases in patients with scabies: A nationwide population-based study in Taiwan. Rheumatol. Int..

[13] Ngo S.T., Steyn F.J., McCombe P.A. (2014). Gender differences in autoimmune disease. Front. Neuroendocrinol..

[14] Adams Waldorf K.M., Nelson J.L. (2008). Autoimmune disease during pregnancy and the microchimerism legacy of pregnancy. Immunol. Investig..

[15] Khashan A.S., Kenny L.C., Laursen T.M., Mahmood U., Mortensen P.B., Henriksen T.B., O’Donoghue K. (2011). Pregnancy and the Risk of Autoimmune Disease. PLoS ONE.

[16] Bergink V., Kushner S.A., Pop V., Kuijpens H., Lambregtse-van den Berg M.P., Drexhage R.C., Wiersinga W., Nolen W.A., Drexhage H.A. (2011). Prevalence of autoimmune thyroid dysfunction in postpartum psychosis. Br. J. Psychiatry.

[17] Lin C.Y., Chang F.W., Yang J.J., Chang C.H., Yeh C.L., Lei W.T., Huang C.F., Liu J.M., Hsu R.J. (2017). Increased risk of bipolar disorder in patients with scabies: A nationwide population-based matched-cohort study. Psychiatry Res..

[18] American Hospital Association, American Medical Record Association, Health Care Financing Administration, National Center for Health Statistics (1990). ICD-9-CM coding and reporting official guidelines. J. Am. Med. Rec. Assoc..

[19] Chen J.Y., Liu J.M., Chang F.W., Chang H., Cheng K.C., Yeh C.L., Wei Y.F., Hsu R.J. (2016). Scabies increased the risk and severity of COPD: A nationwide population-based study. Int. J. Chronic Obstruct. Pulmon. Dis..

[20] Assad S., Khan H.H., Ghazanfar H., Khan Z.H., Mansoor S., Rahman M.A., Rahman M.A., Khan G.H., Zafar B., Tariq U., Malik S.A. (2017). Role of Sex Hormone Levels and Psychological Stress in the Pathogenesis of Autoimmune Diseases. Cureus.

[21] Baillargeon J., Snih S.A., Raji M.A., Urban R.J., Sharma G., Sheffield-Moore M., Lopez D.S., Baillargeon G., Kuo Y.F. (2016). Hypogonadism and the risk of rheumatic autoimmune disease. Clin. Rheumatol..

[22] Fairweather D., Frisancho-Kiss S., Rose N.R. (2008). Sex Differences in Autoimmune Disease from a Pathological Perspective. Am. J. Pathol..

[23] Chatzicharalampous C., Rizos D., Pliatsika P., Leonardou A., Hasiakos D., Zervas I., Alexandrou A., Creatsa M., Konidaris S., Lambrinoudaki I. (2011). Reproductive hormones and postpartum mood disturbances in Greek women. Gynecol. Endocrinol..

[24] Szpunar M.J., Parry B.L. (2018). A systematic review of cortisol, thyroid-stimulating hormone, and prolactin in peripartum women with major depression. Arch. Womens Ment. Health.

[25] Gleicher N. (2007). Postpartum depression, an autoimmune disease?. Autoimmun. Rev..

[26] Toh B.H., van Driel I.R., Gleeson P.A. (1997). Pernicious anemia. N. Engl. J. Med..

[27] Liu J.M., Hsu R.J., Chang F.W., Chiu F.H., Yeh C.L., Huang C.F., Chang S.T., Lee H.C., Chi H., Lin C.Y. (2017). Increased risk of pernicious anemia following scabies: A nationwide population-based matched-cohort study. Ther. Clin. Risk Manag..

[28] Nishiura H., Iwamoto S., Kido M., Aoki N., Maruoka R., Ikeda A., Chiba T., Watanabe N. (2013). Interleukin-21 and tumor necrosis factor-alpha are critical for the development of autoimmune gastritis in mice. J. Gastroenterol. Hepatol..

[29] van der Woude D.A.A., Pijnenborg J.M.A., de Vries J., van Wijk E.M. (2018). The distribution of total vitamin B12, holotranscobalamin, and the active vitamin B12 fraction in the first 5 weeks postpartum. Int. J. Lab. Hematol..

[30] Goshtasebi A., Alizadeh M., Gandevani S.B. (2013). Association between Maternal Anaemia and Postpartum Depression in an Urban Sample of Pregnant Women in Iran. J. Health Popul. Nutr..

[31] Dickens C., McGowan L., Clark-Carter D., Creed F. (2002). Depression in rheumatoid arthritis: A systematic review of the literature with meta-analysis. Psychosom. Med..

[32] Matcham F., Rayner L., Steer S., Hotopf M. (2013). The prevalence of depression in rheumatoid arthritis: A systematic review and meta-analysis. Rheumatology.

[33] Fu X., Li Z.J., Yang C.J., Feng L., Sun L., Yao Y., Huang Y.T. (2017). The prevalence of depression in rheumatoid arthritis in China: A systematic review. Oncotarget.

[34] Le Donne M., Mento C., Settineri S., Antonelli A., Benvenga S. (2017). Postpartum Mood Disorders and Thyroid Autoimmunity. Front. Endocrinol..

[35] Bergink V., Pop V.J.M., Nielsen P.R., Agerbo E., Munk-Olsen T., Liu X. (2018). Comorbidity of autoimmune thyroid disorders and psychiatric disorders during the postpartum period: A Danish nationwide register-based cohort study. Psychol. Med..

[36] Chen H.H., Yeh S.Y., Lin C.L., Chang S.N., Kao C.H. (2014). Increased depression, diabetes and diabetic complications in Graves’ disease patients in Asia. QJM.

[37] Di Bari F., Granese R., Le Donne M., Vita R., Benvenga S. (2017). Autoimmune Abnormalities of Postpartum Thyroid Diseases. Front. Endocrinol..

[38] Wesseloo R., Kamperman A.M., Bergink V., Pop V.J.M. (2018). Thyroid peroxidase antibodies during early gestation and the subsequent risk of first-onset postpartum depression: A prospective cohort study. J. Affect. Disord..

[39] Alexander E.K., Pearce E.N., Brent G.A., Brown R.S., Chen H., Dosiou C., Grobman W.A., Laurberg P., Lazarus J.H., Mandel S.J. (2017). 2017 Guidelines of the American Thyroid Association for the Diagnosis and Management of Thyroid Disease During Pregnancy and the Postpartum. Thyroid.

[40] Martino M., Rocchi G., Escelsior A., Fornaro M. (2012). Immunomodulation Mechanism of Antidepressants: Interactions between Serotonin/Norepinephrine Balance and Th1/Th2 Balance. Curr. Neuropharmacol..

