# Supplementary material for: Postpartum Depression and Subsequent Autoimmune Diseases in Taiwan
Source: Int J Environ Res Public Health. 2018 Aug 20;15(8):1783. doi: 10.3390/ijerph15081783 (PMC6121646; doi:10.3390/ijerph15081783)
Supplement: Supplementary file 1 [file ijerph-15-01783-s001.docx]

**Table S1**. Diseases and corresponding ICD-9-CM codes in the study.

| **Diseases** | **ICD-9-CM code** |
| --- | --- |
| PPD | 296.2, 296.3, 296.5, 296.82, 300.4, 309.0, 309.1, 311, 648.4, 648.44, 780.79 |
| **Autoimmune diseases** |  |
| RA | 714.0 |
| Psoriasis | 696, 696.1 |
| Systemic lupus erythematosus | 710.0 |
| Multiple sclerosis | 340 |
| Graves’ disease | 242, 242.01 |
| Hashimoto’s thyroiditis | 245.2 |
| Ankylosing spondylitis | 720.0 |
| Crohn’s disease | 555.0, 555.1, 555.2, 555.9 |
| Guillain-Barre’ syndrome | 357.0 |
| Sjo¨gren syndrome | 710.2 |
| Myasthenia gravis | 358 |
| Pernicious anemia | 281 |
| Hereditary haemolytic anaemia | 282 |
| Polyarteritis nodosa | 446 |
| Celiac disease | 579 |
| Dermatomyositis | 710.3 |
| Hypersensitivity vasculitis | 446.2, 446.29 |
| Behcet’s disease | 136.1 |
| Polymyositis | 710.4 |
| Alopecia areata | 704.01 |
| Wegener’s granulomatosis | 446.4 |
| Ulcerative colitis | 556.0, 556.6, 556.8, 556.9 |
| Autoimmune haemolytic anaemia | 283 |
| Pemphigus | 694.4 |
| Systemic sclerosis | 710.1 |
| Juvenile rheumatoid arthritis | 714.30, 714.33 |
| Goodpasture syndrome | 446.21 |
| Giant cell arteritis | 446.5 |
| Thromboangitis obliterans | 443.1 |
| Arteritis obliterans | 446.7 |
| Kawasaki disease | 446.1 |
| **Comorbid diseases** |  |
| Diabetes mellitus | 250.00, 250.02, 250.10, 250.12, 250.20, 250.22, 250.30, 250.32, 250.40, 250.42, 250.50, 250.52, 250.60, 250.62, 250.70, 250.72, 250.80, 250.82, 250.90, 250.92 |
| Hypertension | 401-405 |
| Hyperlipidemia | 272 |
| Coronary artery disease | 410-414 |
| Stroke | 430-438 |
| Alcoholism | 291, 303, 305.00-305.03, 571.1, 571.2, 571.3, 790.3, A215 and V11.3 |
| Obesity | 278 |
| Tobacco use disorder | 305.1, 491.0, 491.2, 492.8, 496, 523.6, 649.0, 989.84, and V15.82 |

PPD: postpartum depression, RA: rheumatoid arthritis
